# Early surgical intervention for active thoracic spinal tuberculosis patients with paraparesis and paraplegia

**DOI:** 10.1186/s12891-021-04078-y

**Published:** 2021-02-21

**Authors:** Weiwei Li, Zheng Liu, Xiao Xiao, Zhenchao Xu, Zhicheng Sun, Zhen Zhang, Xiyang Wang

**Affiliations:** 1 Department of Spine Surgery and Orthopaedics, Xiangya Hospital, Central South University, 87#Xiangya Road, 410008 Changsha, Hunan China; 2 Department of Orthopedic, Shaanxi Provincial People’s Hospital, 710068 Xi’an, Shaanxi China; 3 National Clinical Research Center for Geriatric Disorders, Xiangya Hospital, Central South University, Changsha, China; 4 Hunan Engineering Laboratory of Advanced Artificial Osteo-materials, Xiangya Hospital, Central South University, 87#Xiangya Road, 410008 Changsha, Hunan China

## Abstract

**Background:**

To explore the therapeutic effect of early surgical intervention for active thoracic spinal tuberculosis (TB) patients with paraparesis and paraplegia.

**Methods:**

Data on 118 active thoracic spinal TB patients with paraparesis and paraplegia who had undergone surgery at an early stage (within three weeks of paraparesis and paraplegia) from January 2008 to December 2014 were retrospectively analyzed. The operation duration, blood loss, perioperative complication rate, VAS score, ASIA grade and NASCIS score of neurological status rating, Erythrocyte Sedimentation Rate (ESR), C-reactive protein (CRP), kyphotic Cobb’s angle, and duration of bone graft fusion were analyzed to evaluate the therapeutic effects of surgery.

**Results:**

The mean operating time was 194.2 minutes, and the mean blood loss was 871.2 ml. The perioperative complication rate was 5.9 %. The mean preoperative VAS score was 5.3, which significantly decreased to 3.2 after the operation and continued decreasing to 1.1 at follow up (*P*<0.05). All cases achieved an increase of at least one ASIA grade after operation. The rate of full neurological recovery for paraplegia (ASIA grade A and B) was 18.0 % and was significantly lower than the rate (100 %) for paraparesis (ASIA grade C and D) (*P*<0.05). On the NASCIS scale, the difference in the neurological improvement rate between paraplegia (22.2 % ± 14.1 % in sensation and 52.2 % ± 25.8 % in movement) and paraparesis (26.7 % ± 7.5 % in sensation and 59.4 % ± 7.3 % in movement) was remarkable (*P*<0.05). Mean preoperative ESR and CRP were 73.1 mm /h and 82.4 mg/L, respectively, which showed a significant increase after operation (*P*>0.05), then gradually decreased to 11.5 ± 1.8 mm/h and 2.6 ± 0.82 mg/L, respectively, at final follow up (*P*<0.05). The mean preoperative kyphotic Cobb’s angle was 21.9º, which significantly decreased to 6.5º after operation (*P*<0.05) while kyphotic correction was not lost during follow up (*P*>0.05). The mean duration of bone graft fusion was 8.6 ± 1.3 months.

**Conclusions:**

Early surgical intervention may be beneficial for active thoracic spinal TB patients with paraparesis and paraplegia, with surgical intervention being more beneficial for recovery from paraparesis than paraplegia.

## Background

Tuberculosis (TB) is an infectious disease caused by mycobacterium TB (MTB), and its prevalence depends on local economic, hygienic, and medical conditions. TB is found worldwide but is mainly prevalent in developing and undeveloped countries, such as India, China, and certain African countries. According to the global TB report published in 2019, the incidence of TB in developed countries was 24 to 32 per 100,000 individuals, and in developing countries was 35 to 866 per 100,000 individuals [1]. The lung is the organ most frequently affected by MTB. The effect of MTB on the lung may result in a cough, expectoration, thoracalgia, hemoptysis, fever, and symptoms of exhaustion. If the lung lesion is not well controlled, other organs can also be affected. Spinal TB is one of the most common extrapulmonary types of TB and is the result of a primary lung lesion caused by MTB, transmitted through the Batson plexus [2].

Spinal TB can damage the vertebral body, discs, and paravertebral soft tissue, resulting in caseous necrotic tissue, pus, and dead bone, which can enter the spinal canal and cause spinal cord compression, leading to paraparesis and even paraplegia. Kotil et al. [3] reported that most spinal TB patients could be cured using anti-TB drug chemotherapy. However, the blind pursuit of anti-TB drug chemotherapy while neglecting surgical intervention may lead to incurable disability and severe kyphotic deformity. Thus, surgical intervention is recommended for spinal TB patients complicated by spinal instability, spinal cord compression, sequestration, paravertebral abscess, and sinus formation.

According to Velayutham et al. [4], spinal TB is one of the ten most common diseases that cause disability in India. The number of new spinal TB cases in China has been continuously high, resulting in damage to the physical and mental health of patients while placing a huge economic burden on their families and society as a whole. Spinal TB can occur in any part of the spine. For children and adolescents, the thoracic spine is most frequently affected, while for adults, the most frequently affected site is the lumbar spine. Due to the narrow thoracic spinal canal and insufficient blood supply in the thoracic spinal cord, paralysis can quickly occur in patients with thoracic spinal TB if treatment is not administered promptly or if incorrect treatment is administered [5]. Paraparesis and paraplegia are two different phases of neurological deficit. Paraparesis is characterized by impairment of strength, while the loss of strength characterizes paraplegia, and both conditions focus on motor function without an effect on sensory function [6]. The purpose of this study is to investigate the effect of early surgical intervention for active thoracic spinal TB with paraparesis and paraplegia and compared the effect of neurological improvement between paraparesis and paraplegia.

## Materials and methods

Inclusion criteria were: (1) patients with confirmed spinal TB, defined as patients with positive MTB culture or typical TB caseous necrosis and granuloma; (2) surgical intervention was carried out within three weeks of the onset of paraparesis and paraplegia; (3) the follow-up period was more than 12 months. Exclusion criteria were: (1) patients with severely damaged hepatic function; (2) patients who can ambulate smoothly without the aid of a walking stick; (3) patients with other concurrent disorders that may impair motor or sensational function of lower extremities; (4) patients with systemic TB. A total of 118 consecutive patients with confirmed spinal TB and concurrent neurological deficit were enrolled in this study from January 2008 to December 2014. The patients included 75 males and 43 females of an average age of 32.0 ± 9.9 years (14–63 years). The duration of the neurological deficit ranged from 1 to 21 days, with a mean of 6.3 ± 4.9 days. Blood routine (BR), Erythrocyte Sedimentation Rate (ESR), C-reactive protein (CRP), and the T-cell spot test for TB infection were conducted for diagnosis. Pulmonary CT scan, sputum smear, and culture were conducted to exclude active systematic TB patients. X-rays, CT scans, and magnetic resonance (MR) of the affected spinal area were examined to determine the location, severity, and extent of the lesion. The lesion site distribution among the patients was as follows: 24 cases in the upper thoracic (T1-T4), 42 cases in the middle thoracic (T5-T9), and 52 cases in the thoracolumbar (T10-T12). 79 cases involved one diseased function spinal unit (FSU), 25 cases involved two FSUs, and 14 cases involved 3 or more FSUs. The neurological status of the patients based on the American Spinal Cord Injury Association (ASIA) evaluation system was as follows: 14 cases of grade A, 47 cases of grade B, and 57 cases of grade C. Paraparesis is characterized as impairment of strength, while paraplegia is characterized by the loss of strength [7]. In this study, paraparesis cases included 57 ASIA grade C and no ASIA grade D patients, while cases with paraplegia included 14 ASIA grade A and 47 ASIA grade B patients. The National Acute Spinal Cord Injury Study (NASCIS) scale was applied to assess spinal cord functionality [7]. The average NASCIS scores for paraplegia were 78.6 ± 15.2 in sensory function and 50.0 ± 0.0 in motor function before operation. The average NASCIS scores for paraparesis were 88.5 ± 4.9 for sensory function and 70.7 ± 3.5 for motor function before operation. All patients presented with varying degrees of back pain, which lasted from 1 to 28 months, with an average of 8.1 ± 5.3 months. 73 cases (61.9 %) had low afternoon fever, night sweats, fatigue, and other typical TB disease symptoms. All cases presented with paravertebral abscess, vertebral collapse, and sequestra formation, as observed through CT scans. Spinal cord compression was observed in all cases, and no abnormal signal intensity of the spinal cord was observed through the MRI examination. Confirmation of TB infection depended on the postoperative typical histopathological presentation or MTB culture. The mean preoperative Visual Analog Scale (VAS) score was 5.3 ± 1.7 (range, 3–7). The mean preoperative ESR and CRP values were 73.1 ± 12.4 mm/h (range, 30–101 mm/h) and 82.4 ± 15.6 (36–110 mg/L), respectively. The mean preoperative kyphotic Cobb’s angle was 21.9º ± 4.6º (range, 8º-35º). All surgical interventions were administered within 3 weeks of paraparesis or paraplegia. Ethical approval was obtained from the Ethics Committee of Xiangya Hospital affiliated with Central South University, and informed consent was obtained from all patients before undergoing the technique (Table 1).

**Table 1:** Basic clinical data of patients

Patient Characteristics	Value
Gender (male/female)	75/43
Age (years)	32.0 ± 9.9
Lesion location	
T_1 − 4_	24(20.3 %)
T_5 − 9_	42(35.6 %)
T_10 − 12_	52(44.1 %)
Number of affected FSUs	
single	79(66.9 %)
double	25(21.2 %)
Multiple	14(11.9 %)
Duration for back pain	8.1 ± 5.3
Paraparesis or paraplegia duration (days)	6.3 ± 4.9
Operation time (min)	194.2 ± 48.2
Blood loss	871.2 ± 161.2
Rate of perioperative complication	7 (5.9 %)
Mean duration of bone graft fusion	8.6 ± 1.3

### Preparation for surgery

All patients received standard quadruple anti-TB drug therapy (0.3 g of Isoniazid, 0.45 g of Rifampicin, 0.75 g of Pyrazinamide, and 0.75 g of Ethambutol per day) combined with antibiotics, levofloxacin (0.4 g per day), and streptomycin (0.57 g per day). The duration of the anti-TB drug treatment was at least two weeks if the neurological function of the patient did not deteriorate. However, if the neurological status was deteriorating, emergency surgery was carried out immediately. Anemia and hypoproteinemia were treated before surgery. Cefuroxime (intravenous drip, 1.5 g) or Cefazolin (intravenous drip, 1.5 g) was administered 30 minutes before surgery.

### Procedure

**Fig. 1:**
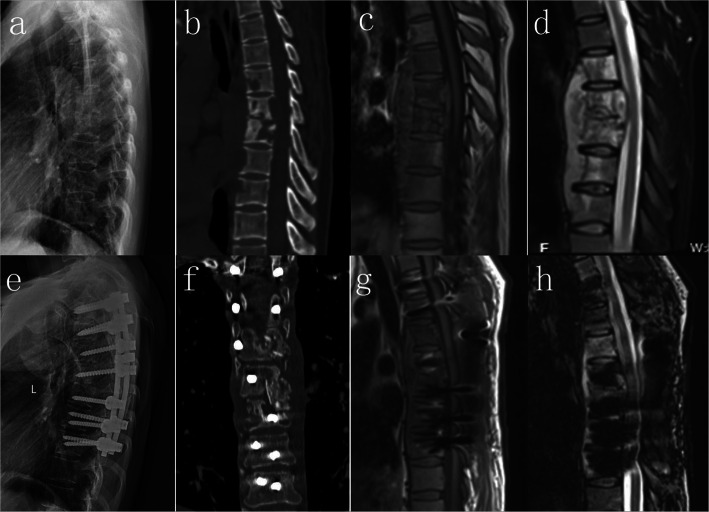
A 33 year old male patient with T6-7 active spinal TB and paraparesis received one stage posterior spinal cord decompression, lesion removal, interbody fusion and pedicle fixation. (**a**-**d**). Preoperative images show cavity formation of vertebral body, disc necrosis, paraspinal and epidural cold abscess formation. (**e**-**h**) Postoperative image within 7 days after the operation show most paraspinal and epidural cold abscesses were removed through one stage posterior surgery, and good position of pedicle screw and interbody fusion was obtained

**Fig. 2:**
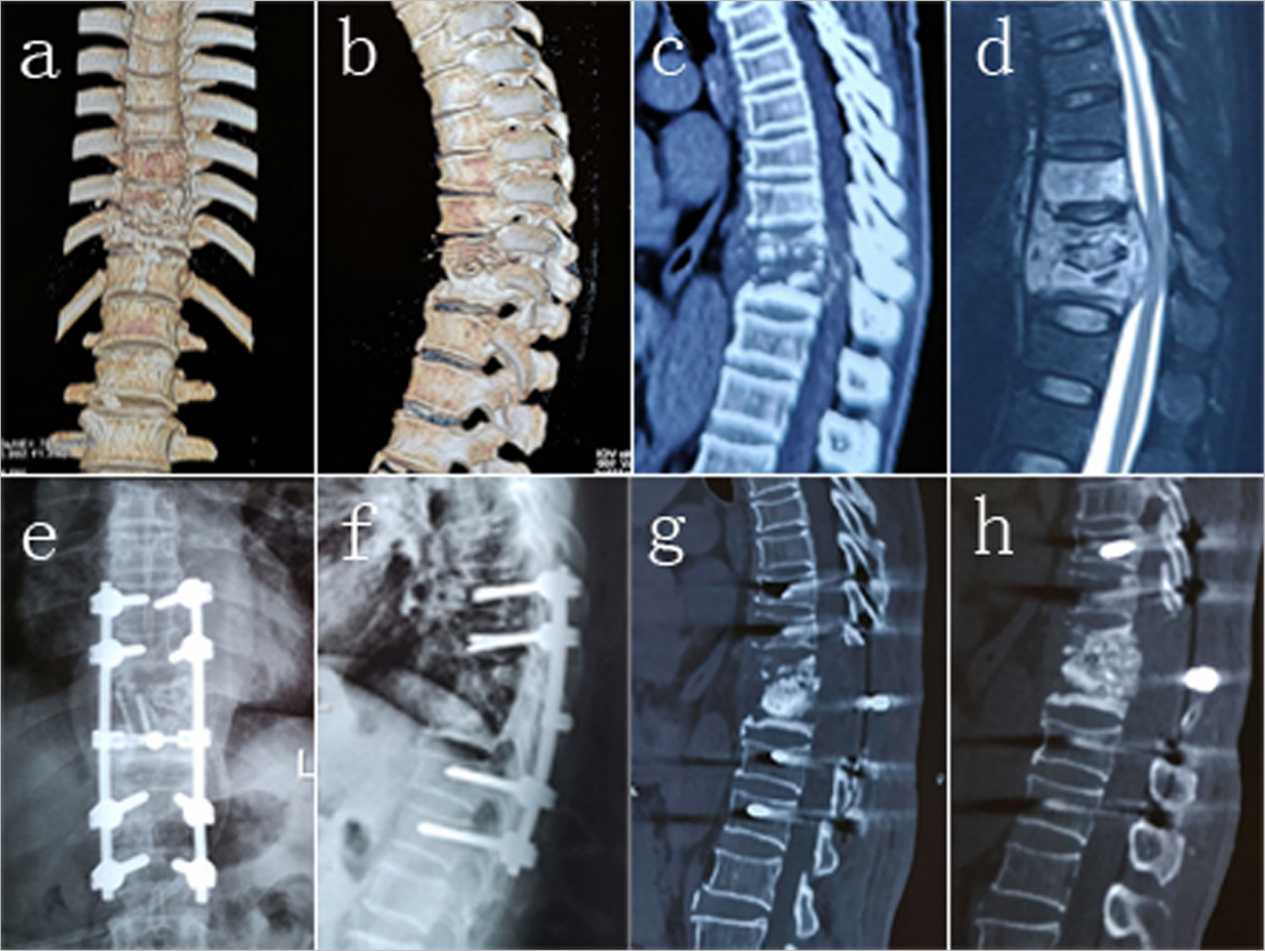
A 46 year old male patient with T10-11 active spinal TB and paraplegia received one stage posterior spinal cord decompression, lesion removal, interbody fusion and pedicle fixation. (**a**–**d**), Preoperative images show osteolytic destruction and collapse of vertebral body, epidural cold abscess formation and spinal cord compression. (**e**-**g**) Postoperative image within 7 days after the operation show good position of pedicle screw fixation and interbody bone graft, as well as correction of kyphosis deformity. (**h**) Postoperative image 12 months after the operation shows that interbody fusion was achieved and that no significant loss of the kyphotic correction was observed

One stage posterior spinal cord decompression, lesion removal, interbody fusion, pedicle fixation, or posterior spinal cord decompression, pedicle fixation combined with two-stage anterior debridement, and interbody fusion were performed based on the location of the lesion and size of the paravertebral abscess. The key technical steps followed when performing one-stage posterior surgery were as follows: pedicle screws were placed in the normal vertebras, and the fixation range included the diseased area and 1–2 upper and lower normal FSUs; laminectomy and pedicle resection were performed to facilitate spinal cord decompression and removal of necrotic discs, caseous abscess, and sequestra. The paravertebral abscesses in the unseen view were flushed and drained using a catheter. An iliac allograft was placed in the intervertebral space, and autogenous bone fragments from normal spinous processes and laminas were used for the interbody fusion of the remaining intervertebral spaces. The key technical steps performed for posterior spinal cord decompression, pedicle fixation combined with two-stage anterior debridement, and interbody fusion were as follows: the fundamental techniques used for the posterior approach were similar to that of the single posterior surgery mentioned above, without pedicle resection and interbody grafts. The two-stage anterior approach was implemented one week after the posterior approach. Single lung ventilation was used during operation, and the lesion was accessed via an extrapleural or transthoracic approach. An iliac allograft block was placed in the intervertebral space after complete debridement, and closed chest drainage was performed at the end of the surgery (Figs. 1 and 2). The operation duration and intraoperative blood loss were noted to determine the surgical effect.

### Postoperative care

Temperature and lower extremity movements were closely monitored. The same anti-infection protocol was used for 24–48 h. The drainage tube was removed after the volume of drainage was less than 20 ml/24 h. Mild activity out of bed was advocated with the protection of a thoracolumbar brace after drainage removal. The perioperative complication rate was noted to evaluate surgical results. The amount of physical activity was gradually increased, and the brace support was used for at least 3 months. The anti-TB protocol followed during the first 4 postoperative weeks after operation was the same as the preoperative anti-TB protocol. The subsequent anti-TB scheme used was standard quadruple anti-TB drug therapy for 12 to 18 months.

### Follow‐up and efficacy evaluative indexes

Follow-up visits were mainly conducted at outpatient clinics. The follow-up indices included symptoms (fever, night sweats, fatigue, and VAS score for back pain), neurological status (ASIA grade and NASCIS scale), clinical lab index (ESR and CRP), kyphotic Cobb’s angle, and time of bone graft fusion. The follow-up interval was every 3 months within the first year after operation, every 6 months within the second year after operation, and once a year from the second year after operation.

## Results

All patients were followed up for at least 12 months, with an average follow-up duration of 28.0 ± 10.0 months (range, 12–60 months). No perioperative TB systematic dissemination and mortality was observed in any of the patients during hospitalization or the subsequent follow-up period. All surgeries were carried out successfully without intraoperative iatrogenic neural and vascular injury. 92 patients (92/118, 78 %) underwent one-stage posterior surgery, and 26 patients (26/118, 22 %) underwent posterior decompression and fixation combined with two-stage anterior debridement and interbody fusion. Two patients underwent revision surgery of anterior debridement due to the prolonged existence of paravertebral abscess after a single posterior approach (2/92, 2.2 %). All other patients healed smoothly after the single-stage approach. The mean duration of surgery was 194.2 ± 48.2 min (range, 120–360 min), and mean blood loss was 871.2 ± 161.2 ml (range, 600–1500 ml) (Table 1). During the hospitalization period, one case was complicated with pleural rupture. Five cases were complicated with a dura tear and cerebrospinal fluid leak, while one case was complicated with superficial wound infection, resulting in a perioperative complication rate of 5.9 % (Table 1). All symptoms that are characteristic of TB disease disappeared 3 months after operation. The VAS score of back pain improved significantly after operation (3.2 ± 1.3, range 2–4) (*P*<0.05), and continued to improve, the mean VAS score at final follow-up was 1.1 ± 0.53 (range 0–3), which significantly lower than the postoperative score (*P*<0.05). Improvement of the neurological status from pre-operation to the final follow-up was as follows: 2 patients with ASIA grade A changed to grade B, 10 patients with ASIA grade A changed to grade D, 2 patients with ASIA grade A changed to grade E; 38 patients with ASIA grade B changed to grade D, 9 patients with ASIA grade B changed to grade E; all patients with ASIA grade C changed to ASIA grade E (Table 2). The full neurological recovery rate for paraplegia (ASIA grade A and grade B) was 18.0 %, while the full neurological recovery rate for paraparesis (ASIA grade C and D) was 100 %. The difference in full neurological function recovery rate between the patients with paraplegia and paraparesis was significant (*P*<0.05). The average NASCIS scores for patients with paraplegia was 100.5 ± 8.2 for sensation, and 77.5 ± 12.4 for movement at the final follow up. The average NASCIS scores for patients with paraparesis was 112.0 ± 0.0 for sensation and 100.0 ± 0.0 for movement at the final follow up. Using the NASCIS scale, neurological improvement can be denoted using the following formula: the neurological improvement rate = (postoperative NASCIS score- preoperative NASCIS score) / preoperative score. The difference in neurological improvement rate using NASCIS scale system between patients with paraplegia (improvement rate for sensation and movement were 22.2 % ± 14.1 % and 52.2 % ± 25.8 %, respectively) and patients with paraparesis (improvement rate for sensation and movement were 26.7 % ± 7.5 % and 59.4 % ± 7.3 %, respectively) was significant. (*P*<0.05) (Table 3). The value of ESR and CRP were 74.8 ± 10.8 mm/h and 83.6 ± 13.4 mg/L after operation, which is not significantly higher than the corresponding preoperative indexes (*P*>0.05) and decreased gradually to the normal level within 6 months. The mean ESR and CRP values at the final follow-up were 11.5 ± 1.8 mm/h and 2.6 ± 0.82 mg/L, respectively, significantly lower than corresponding preoperative indexes (*P*<0.05) (Table 4). Postoperatively, kyphotic Cobb’s angle 6.5º±1.5º (range, 0º-12º) was found to have been significantly corrected (*P*<0.05), compared with the preoperative status, and the correction was not lost during the follow-up period (*P*>0.05) (Table 4). The mean duration of bone graft fusion was 8.6 ± 1.3 months (range, 6–14 months). (Table 1).

**Table 2:** ASIA grade for neurological status assessment

Preoperative			Final follow-up				
			A	B	C	D	E
Paragplegia	A	14		2		10	2
	B	47				38	9
Paraparesis	C	57					57
	D

**Table 3:** NASCIS score for neurological status assessment and improvement rate comparsion

NASCIS score		Preoperation	Final follow up	Neurological improvement rate
Paragplegia	Sensation	78.6 ± 15.2	100.5 ± 8.2	22.2 % ± 14.1 %
	Movement	50.0 ± 0.0	77.5 ± 12.4	52.2 % ± 25.8 %
Paraparesis	Sensation	88.5 ± 4.9	112.0 ± 0.0	26.7 % ± 7.5 %*
	Movement	70.7 ± 3.5	100.0 ± 0.0	59.4 % ± 7.3 %Δ

The neurological improvement rate = (postoperative NASCIS score- preoperative NASCIS score) / preoperative NASCIS score

*P < 0.05 vs. Comparison of sensation improvement rate of between paraplegia and Paraparesis

Δ*P* < 0.05 vs. Comparison of movement improvement rate between paraplegia and Paraparesis

**Table 4:** Preoperative, postoperation, and final follow-up indexes of VAS ESR, CRP and Kyphotic Cobb’s angle

Observational indexes	Preoperation	Postoperation	Final follow up
VAS	5.3 ± 1.7	3.2 ± 1.3*	1.1 ± 0.53*Δ
ESR (mm/h)	73.1 ± 12.4	74.8 ± 10.8	11.5 ± 1.8*Δ
CRP (mg/L)	82.4 ± 15.6	83.6 ± 13.4	2.6 ± 0.82*Δ
Kyphotic Cobb’s angle (º)	21.9 ± 4.6	6.5 ± 1.5*	6.6 ± 1.9*

*P < 0.05 vs. Preoperation

Δ*P* < 0.05 vs. Postoperation

## Discussion

Neurological deficit is the most severe spinal TB complication, mainly due to direct mechanical spinal cord compression due to an expanding abscess, caseous necrotic material, TB granulation tissue, and bony elements [8]. Other mechanisms, such as instability, meningitis, infective thrombosis of spinal vessels, and some adverse changes in the spinal cord, have also been implicated as causes of the degeneration of neurological status [9]. Paraparesis is described as impairment of strength or weakness in spinal cord function, while paraplegia is defined as the loss of motor function of the spinal cord [6]. Barcelos et al. [6] have stated that based on the well-established definition, complete paraplegia should not only include ASIA grade A but should also include ASIA grade B because the definition of paraplegia is based on motor strength loss, without sensory function assessment. Similarly, paraparesis should include both ASIA grade C and D because the definition of paraparesis also emphasizes impairment of motor function while ignoring sensory function.

The classification of paraparesis and paraplegia in spinal TB includes early-onset paraparesis and paraplegia (active spinal TB) and late-onset paraparesis and paraplegia (healed spinal TB), as proposed by Hodgson et al. [10]. Early-onset paraparesis and paraplegia for spinal TB are mainly caused by soft pressure (pus, caseous necrotic tissue, and tuberculous granuloma) on the spinal cord resulting in slow, continuous, and gradual compression. Late-onset paraparesis and paraplegia for spinal TB are mainly caused by rigid pressure (kyphotic bone ridge, granulomatous scar, and contracture) [11]. All cases included in our study were of patients with early-onset paraparesis and paraplegia, as determined through radiological presentation and operation findings. The materials that had caused spinal cord compression among patients included in our study were primarily soft materials, such as fluid pus, proliferous granuloma, and osteolytic bony destruction. Therefore, this finding is consistent with that of previous literature reports. Since spinal cord disturbance was only exerted for a short duration, no signals of spinal cord denaturation, necrosis, and malacia were detected through MRI examinations. However, epidural adhesion was found in most patients, resulting in a higher rate of dural tears and cerebrospinal fluid leak.

Paraparesis and paraplegia as a result of spinal TB can be cured or improved. Some researchers have suggested that early onset paraparesis and paraplegia can be cured using conservative anti-TB drug management because only soft pressure has been exerted on the spinal cord and can be easily absorbed under effective anti-TB drug treatment [12]. However, many experts have mentioned that irreparable damage to neurological function may occur due to long periods of anti-TB drug conservative treatment. Therefore, surgical intervention needs to be administered only when necessary [13, 14]. For our series of patients, a minimum period of 2 weeks of anti-TB drug therapy was usually administered. However, for patients with a drastically deteriorating neurological status, the duration of preoperative anti-TB drug therapy was much less than 2 weeks due to the need for emergency surgery.

The objective of surgical intervention is to effectively and safely relieve neural pressure, maximizing the decrease of the infectious burden, and reconstructing spinal stability, while minimizing physical damage. Until now, the timing suitable for surgical intervention for paraparesis and paraplegia of spinal TB has been disputed [15]. Batirel et al. [16] proposed that paraplegia caused by spinal TB is a slowly developing process. A short delay in surgical decompression may not change the final level of recovery of neurological function. Wang et al. [17] and Chandra et al. [18] advocated for 2–4 weeks of standard quadruple anti-TB drug treatment before operation but stated that emergency operations might be needed as neurological impairment progresses. Zhang et al. [19] stated that surgery could be performed after a significant decrease in ESR and CRP has been observed. Jia et al. [20] conducted a study that compared early (< 4 weeks) and non-early surgery (≥ 4 weeks) for the treatment of spinal TB with neurological deficit. They recommended early surgical management due to the superior improvement of neurological status in the early surgery group.

For our series, the early operation was defined as an operation carried out within 3 weeks of paraparesis and paraplegia. Before operation, a standard quadruple anti-TB drug therapy combined with the use of levofloxacin and streptomycin were administered to prevent the dissemination of TB. In contrast, the postoperative anti-TB regimen was usually standard quadruple anti-TB drug therapy if the hepatic function was not severely damaged or drug resistance was not encountered. The total duration of the anti-TB therapy was 12–20 months. We found significant neurological function improvement together with other positive indexes after early surgery. However, a differentiated result for full neurological recovery rate was observed between the paraparesis (100 %) and paraplegia (18.0 %). Thus, in our opinion, early surgical intervention is more beneficial for the neurological recovery of spinal TB patients with paraparesis than with paraplegia.

Some experts have been concerned that early surgical intervention may lead to the dissemination of systemic TB. In our series, the elevated ESR and CRP values were transiently not significant after surgery. However, no cases of disseminated TB were observed during the hospitalization period and subsequent follow-up. The anterior approach is commonly used for spinal TB and has some merit for debridement and interbody fusion. However, some authors, including our researchers, have reported that it is less accessible for spinal canal decompression [21]. Li et al. [22] reported that single anterior debridement decompression, autogenous rib grafts, and instrumentation are beneficial for spinal TB patients. Varatharajah et al. [23] reported that anterior surgery is beneficial for debridement and kyphosis correction but may result in low maintainability of kyphosis correction.

In recent years, many researchers have reported successful results from single posterior surgery for spinal TB. For patients with spinal TB with paraparesis or paraplegia, spinal cord decompression is more accessible through a posterior approach. Thus the single posterior approach is a suitable choice for spinal TB cases with paraparesis or paraplegia with small prevertebral abscesses [24]. Ukunda et al. [25] found that the posterior-only surgical approach is advantageous for kyphosis correction and disability improvement. Zhang et al. [26] reported that posterior debridement, fixation, and interbody fusion are safe and effective methods for patients with upper thoracic spinal TB. In our series, most patients (92/118,78 %) received a single posterior approach, and the relapse rate was very low (2.2 %). Therefore, the therapeutic effect of the single posterior approach for spinal TB paraparesis and paraplegia was found to be excellent. For cases with large prevertebral abscesses, the procedure of posterior decompression and fixation combined with two-stage anterior debridement and interbody fusion was chosen, and no cases of relapse were reported during the follow-up period. Therefore, we believe that a preoperative CT scan or MRI is essential for choosing the appropriate method of surgery. Additionally, we found that a postoperative CT scan or MRI was also crucial for deciding whether two-stage anterior surgery is necessary or not after the first posterior surgery. If the paravertebral pus has not been cleared and drained well after single posterior surgery, then an additional two-stage anterior debridement was advocated due to decreased probability of TB relapse and faster healing of TB lesions [27, 28].

Kyphotic correction is also an essential index for therapeutic evaluation, and in this study, significant correction of kyphotic Cobb’s angle was achieved after operation. Traditionally, an autograft is the gold standard for bone defect repair and has always been used for interbody fusion. However, an allogeneic bone was used for every patient included in this study due to the reasons listed below. First, allogeneic bone is easily obtained, and it is a mature technique of processing. Second, all patients were willing to accept the allograft and were reluctant to undergo autogenous iliac extraction. Third, the structural allograft can provide instant and robust support strength for interbody fusion and provides less of an implant subsidence risk compared to a mesh or cage. The allograft also has its inherent flaws, such as the lack of osteogenesis, the possibility of graft resorption, and other disadvantages. Some experts [29] are concerned that the use of allograft for interbody fusion may be accompanied by a higher rate of kyphosis correction loss. We observed that correction was not significantly lost, and good alignment was maintained during the follow-up period. This may be due to insufficient observation time (12–60 months, mean was 28.0 months), reservation of the nail and rod fixation system, and the most important fact is that many autogenous bone particles around the allograft were impacted. Therefore, early surgical intervention not only results in faster neurological recovery but can also achieve and sustain excellent kyphotic correction.

This study has some innate limitations. First, it does not include any control cases since it was not a prospective study. Second, the sample size was not large enough. Third, it was not a double-blind study. Therefore, many subjective factors, such as selection bias, may interfere with the results and conclusions. Thus, the conclusions of this study need to be verified using further studies.

## Conclusions

Early surgical intervention may be beneficial for active thoracic spinal TB patients with paraparesis and paraplegia, with surgical intervention being more beneficial for recovery from paraparesis than paraplegia.

## Data Availability

The datasets supporting the conclusions of this article are included within the article. The raw data can be requested from the corresponding author on reasonable request.

## Abbreviations

TB: Tuberculosis
MTB: :Mycobacterium TB
BR: Blood Routine
ESR: Erythrocyte Sedimentation Rate
CRP: C-reactive protein
MR: Magnetic Resonance
FSU: Function Spinal Unit
ASIA: American Spinal Cord Injury Association
NASCIS: National Acute Spinal Cord Injury Study
VAS: Visual Analog Scale
